# A Systematic Review of Associations between Energy Use, Fuel Poverty, Energy Efficiency Improvements and Health

**DOI:** 10.3390/ijerph19127393

**Published:** 2022-06-16

**Authors:** Chengju Wang, Juan Wang, Dan Norbäck

**Affiliations:** Department of Medical Sciences, Occupational and Environmental Medicine, Uppsala University, 75185 Uppsala, Sweden; dan.norback@medsci.uu.se

**Keywords:** health, asthma, respiratory, indoor air quality, built environment, energy use, energy efficiency buildings, green buildings

## Abstract

Energy use in buildings can influence the indoor environment. Studies on green buildings, energy saving measures, energy use, fuel poverty, and ventilation have been reviewed, following the guidelines of the Preferred Reporting Items for Systematic Reviews and Meta-Analyses (PRISMA) statement. The database PubMed was searched for articles published up to 1 October 2020. In total, 68 relevant peer-reviewed epidemiological or exposure studies on radon, biological agents, and chemicals were included. The main aim was to assess current knowledge on how energy saving measures and energy use can influence health. The included studies concluded that buildings classified as green buildings can improve health. More efficient heating and increased thermal insulation can improve health in homes experiencing fuel poverty. However, energy-saving measures in airtight buildings and thermal insulation without installation of mechanical ventilation can impair health. Energy efficiency retrofits can increase indoor radon which can cause lung cancer. Installation of a mechanical ventilation systems can solve many of the negative effects linked to airtight buildings and energy efficiency retrofits. However, higher ventilation flow can increase the indoor exposure to outdoor air pollutants in areas with high levels of outdoor air pollution. Finally, future research needs concerning energy aspects of buildings and health were identified.

## 1. Introduction

In modern society, people spend more than 90% of their time in indoor environments, and most of that time is spent at home [1]. Energy is needed to heat or cool buildings, and energy use in buildings is an important issue in contemporary society [2]. The climate change issue, linked to increased greenhouse gases emissions from coal, oil, or gas combustion, has increased the demand to save energy in buildings in different parts of the world [3]. Because of this demand, different measures have been applied to increase energy efficiency in buildings in order to create a sustainable built environment which combines a healthy and energy-efficient indoor environment [4].

There are three main principles of energy efficiency improvements in buildings: reduced energy use, reduced heat transfer, and reduced air leakage [5]. Reduced energy use can reduce emissions and fuel cost, thus reducing exposure to emissions [6]. Furthermore, reduced heat transfer can increase indoor temperature and reduce relative humidity and risk of mould [7]. In contrast, reduced air leakage can increase relative humidity and risk of mould [8]. In practice, common energy saving measures in buildings include increased thermal insulation, installation of central heating or space heating, draught proofing or installation of heat recovery systems [9]. Since energy use in buildings is a complex issue, scientists from many disciplines, as well as stake holders, government officers, and other decision makers need to work together to make updated energy policies [10].

In recent years, there has been an increase of energy-related labelling of buildings, e.g., low energy buildings, zero energy buildings, green buildings, and healthy buildings [11,12,13]. Green building rating systems have been widely used globally for many years [13]. In the USA, they have created Leadership in Energy and Environmental-Design (LEED) credits to assess green buildings [12]. Other existing green rating systems include BREEAM, CASBEE, Green Star, Enterprise Green Communities, RELi, SITES, Fitwel, Living Building Challenge (LBC), and WELL [11].

However, it should be realized that extreme cold in homes in winter can increase cold-related mortality or morbidity rates [14]. In the UK, fuel poverty is definite as people don’t have enough money to heat their home in winter to maintain an acceptable temperature [15]. However, there is also a cost for cooling their homes in extreme heat situations which some people cannot afford [16].

This systematic review included all types of health aspects of energy use, energy saving, and energy efficiency in buildings. The main aim was to summarize the current knowledge on the health impacts of energy saving measures and energy use. The second aim was to collect knowledge on the indoor environment effects of energy-saving measures and energy use. The third aim was to gather knowledge on types of energy saving or energy use that should be promoted from a health perspective.

## 2. Methods

The guidelines of the Preferred Reporting Items for Systematic Reviews and Meta-Analyses (PRISMA) statement were followed to perform this systematic review [17]. In October 2020, a systematic literature search in PubMed covering articles up to 1 October 2020 was performed. There were ten medical search terms: morbidity, mortality, respiratory, lung function, asthma, rhinitis, eczema, dermatitis, sick building syndrome, building related illness. These medical search terms were combined (any of the ten search terms). In addition, there were eight energy and building related search terms: energy saving, energy use building, energy efficiency building, energy consumption building, energy efficient building, low energy building, energy retrofit, green building. These building and energy-related medical search terms were combined (any of the eight search terms). Then a systematic database search combining any of the ten medical search terms with any of the eight energy and building related search terms was performed. Any medical search term means OR between each search term. Any energy or building related search term means OR between each search term. Combined means AND between the two groups of search terms.

In total, 5776 records were identified from the database searching. Those records were sent to EndNote citation manager for collecting, storing, and organizing. In this reference management software, three reference groups (duplicated group, included group, excluded group) were created. First, 806 duplicated records were removed and added into duplicated group before screening by using the function of EndNote. Then the titles and the abstracts of 4970 articles were screened, to identify articles relevant to the topic of this literature review.

The following three selection criteria were used to include studies in this review:

1. The articles should have studied associations between energy aspects in buildings and health;
2. The articles should be written in English;
3. The articles should not be keynotes, opinions, commentaries, reviews, or modelling studies.

**Figure 1:**
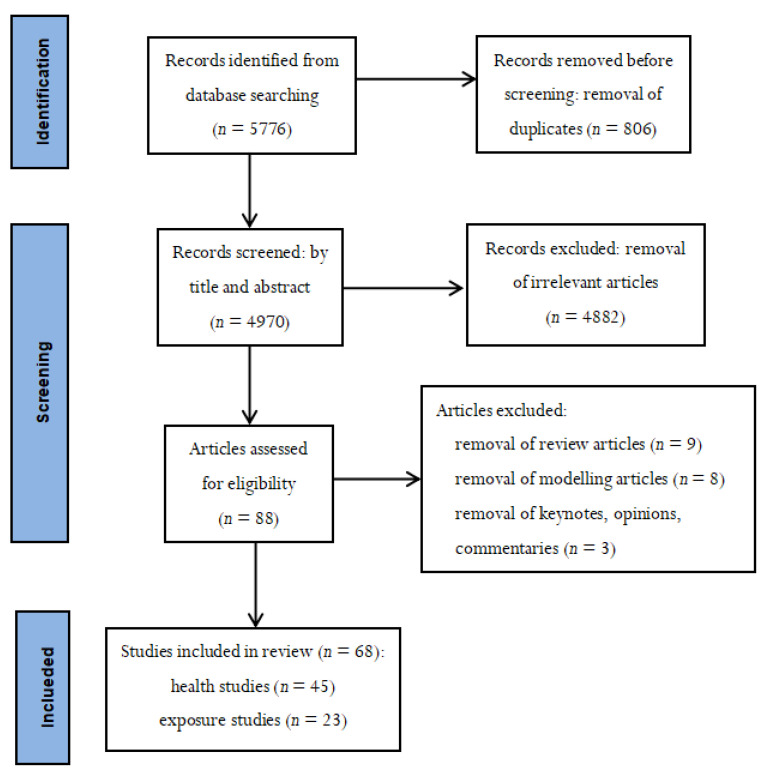
PRISMA flow diagram of literature research.

In total, 4882 irrelevant articles were removed. After removal of irrelevant articles, 88 relevant articles were identified. As a next step, keynotes, opinions, commentaries, review articles and modelling articles were removed. Finally, 68 relevant field studies were included in this review, of which 45 were health studies and 23 studies had measured exposure in relation to energy aspects in buildings without investigating health associations. The PRISMA flow diagram of the literature research is shown in Figure 1.

For each health study, study characteristics on author, year, country, energy aspects, type of study, type of buildings, type of health variables, number of buildings, number of subjects, and main results were extracted. For each exposure study, study characteristics on author, year, country, energy aspects, type of study, type of buildings, measured exposure, changes of measured exposure, number of households, or buildings and main results were extracted. In addition, within the health studies, articles with positive and negative health associations were grouped.

In order to further organize the structure of tables, a thematic classification was made. The studies were divided into four categories: exposure studies, green building health studies, fuel poverty health studies, other energy-related health studies. The exposure studies were divided into three exposure groups, including exposure to radon, exposure to biological agents (mould, bacteria, and house dust mites) and exposure to chemicals. The fuel poverty health studies were divided into three health aspects, including respiratory symptoms, general and mental health, and studies on mortality. Other energy-related health studies were divided into cross-sectional heath studies, longitudinal studies, and intervention health studies according to the study design. Details on those thematic tables can be seen in the Appendix A.

The entire process above involved at least two authors to conduct searching to gathering, screening, analyzing, and extracting.

## 3. Results

### 3.1. Exposure Studies

In Table 1, associations between energy-related building factors and indoor pollutants among the 23 included exposure studies are summarized. These included 19 studies conducted in Europe, 3 studies conducted in USA, and 1 study conducted in China. Except for one school study [18], 22 exposure studies were conducted in residential buildings (Appendix A).

#### 3.1.1. Radon

There were 11 exposure studies on radon [19,20,21,22,23,24,25,26,27,28,29] (Table A1). Of these, 9 studies reported that energy efficiency thermal retrofitting in homes increased radon concentration [19,20,21,22,23,24,25,27,29]. Of these 9 studies, 3 combined thermal insulation with additional air sealing methods in windows [21,22,27]. There were 6 studies of the 9, in five countries, which reported average radon concentrations above 100 Bq/m^3^ in rooms [19,20,22,24,25,27]. However, three studies of 11 demonstrated that energy efficiency retrofitting in homes with installation of mechanical ventilation or other measures can reduce radon concentration [26,27,28]. Other measures included installation of ground covers [26,27] and sub-slab or sump depressurization systems [26].

#### 3.1.2. Biological Agents

There were 8 exposure studies on biological agents [28,29,30,31,32,33,34,35] (Table A2). One study reported that installation of insulated windows and central heating systems increased the concentration of the house dust mites and mould [30]. Another study showed that fuel poverty can increase indoor dampness and mould, regardless of the use of extractor fans [31]. The negative effects may be caused by reduced ventilation [30] and ineffective heating [31]. However, 6 studies of 8 found that energy efficiency improvement in homes with improved ventilation can reduce indoor exposure to mould [28,29,32,34], bacteria [29] and house dust mites [33].

#### 3.1.3. Chemical Substances and Particles

There were 9 exposure studies on chemical substances and particles (Table A3). 4 studies demonstrated that home energy efficiency retrofit can increase indoor air concentrations of certain volatile organic compounds [29,36,38,39] and carbon dioxide levels (CO_2_) [39]. CO_2_ is an indicator of ventilation flow rate. Those volatile organic compounds included formaldehyde [38,39], aromatics [39], alkanes [39] and alpha-pinene [36], hexaldehyde [36], as well as benzene, toluene, ethyl benzene, and xylene (BTEX) [29]. Alpha-pinene and hexaldehyde could be caused by the use of wood or wood-based products for construction and insulation [36]. However, some studies reported that home energy efficiency improvement combined with mechanical ventilation system can reduce aldehydes [28], formaldehyde [29], total volatile organic compounds (TVOC) [28], CO_2_ [18,28,37], carbon monoxide (CO) [27], and black carbon level [38]. One study found that an energy intervention replacing low-polluting semigasifier cooking stoves in rural buildings was associated with decreased exposures to 2.5 (PM_2.5_) particulate matter and black carbon in winter but higher exposure in summer. The negative effect could be caused by increased use of the cooking stove [40].

### 3.2. Health Studies

In Table 2, associations between one kind of fuel poverty, improved ventilation, and energy efficiency improvements and health are summarized. There were 28 studies which were conducted in Europe, 10 studies conducted in the USA, and 7 in other countries, including New Zealand (*n* = 3), Japan (*n* = 2), Canada (*n* = 1), and India, (*n* = 1). Except for three office [41,42,43] and three school studies [44,45,46], 39 studies were performed in residential buildings (Appendix A).

#### 3.2.1. Green Building Health Studies

The green building health studies were conducted in United States (*n* = 5), Canada (*n* = 1), and India (*n* = 1). They were performed in two offices [41,42], two schools [44,45] and three residential buildings (Table A4). Some studies demonstrated that green buildings can reduce self-reported asthma [41,47,48], non-asthmatic respiratory symptoms [41,48], and improve general health [44,45,48,49] and mental health [41,49] as well as performance [41,44,45] and satisfaction [44,45]. One study found no significant association between green buildings and sick building syndrome symptoms (SBS) [42]. Sick building syndrome symptoms include nonspecific symptoms from eyes, skin, upper airways, headache, and fatigue [1].

#### 3.2.2. Fuel Poverty Studies

The fuel poverty studies were conducted in the United Kingdom (*n* = 10), the USA (*n* = 3), New Zealand (*n* = 3), Spain (*n* = 2), Japan (*n* = 1), and multiple countries (*n* = 1). All of the 20 studies were conducted in residential buildings (Table A5, Table A6 and Table A7). Some studies reported that fuel poverty in low-income homes can increase asthma [52] and respiratory symptoms [50,51] and reduce general health [57] and mental health [57]. Furthermore, low indoor air temperature in low-income homes can increase blood pressure [64,67] and hypertension [67] (linked to cold-related mortality). Besides, lack of insulation [65] and heating systems [68] in low-income homes can increase cold-related mortality. However, one study showed that wearable telemetry (a thermometer with a low-temperature alarm) can raise awareness of the health effects of cold living environments among people living in fuel poverty (linked to psychosocial outcomes) [63].

There were another some studies on the effects of improved ventilation or energy efficiency improvements in low-income homes and health. First, they found that high ventilation rates in low-income urban homes may increase chronic cough, asthma, and asthma-like symptoms, probably caused by infiltration of outdoor air pollutants [54]. However, high infiltration rates in low-income, urban, non-smoking homes can improve lung health [85]. Second, they demonstrated that installation of cavity wall insulation in social housing without installation of mechanical ventilation can reduce general health outcomes and social outcomes [53]. Energy efficient façade insulation retrofits in public housing can reduce cold-related mortality in women, but can increase cold-related mortality in men. The reason for the gender difference is unclear [66]. However, energy efficiency improvements in low-income homes can improve respiratory symptoms [53,55,56], general health [53,55,58,59,60] and mental health [53,58] as well as psychosocial outcomes [53,61,62], well-being [55,59,61,62], and sleep [58].

#### 3.2.3. Cross-Sectional Health Studies

The cross-sectional health studies were conducted in Sweden (*n* = 4), the United Kingdom (*n* = 2), Norway (*n* = 1), and Germany (*n* = 1). Expect for one school study [46], seven studies were performed in residential buildings (Table A8). Some studies investigated the association between ventilation and health. They reported that higher ventilation rate in homes were associated with less asthma symptoms [72,73]. Furthermore, in multi-family buildings, lack of a mechanical ventilation system was associated with increased prevalence of SBS-related symptoms [69]. Further, buildings with balanced ventilation systems (supply/exhaust ventilation) had a higher prevalence of doctor diagnosed allergies, as compared to buildings with exhaust ventilation only [71].

There were some other investigative studies on the health impacts of energy efficiency in buildings. First, they found that air tightness [69,74] and use of direct electric radiators [69] in residential buildings were associated with increased prevalence of SBS-related symptoms. However, higher insulation level in buildings was associated with less SBS symptoms [70]. Second, buildings using more energy for heating were associated with lower rates of pollen allergies and eczema [71]. Energy efficiency improvements by boiler replacements in homes were associated with less admission rates for asthma and chronic obstructive pulmonary disease (COPD) [73]. Third, lower air temperature in buildings at a university campus was associated with less tear film stability [46]. Higher thermal variety (linked to lower domestic demand temperatures) was associated with fewer morbidities related to cold mortality [75].

#### 3.2.4. Longitudinal Health Studies

A longitudinal study from Austria found that energy efficient buildings combined with installation of mechanical ventilation can improve general health and mental health but increase dry eye symptoms, as compared to conventional buildings with natural ventilation only [76] (Table A9).

#### 3.2.5. Intervention Health Studies

Intervention health studies were conducted in the United Kingdom (*n* = 3), the United States (*n* = 2), Japan (*n* = 1), Sweden (*n* = 1), Denmark (*n* = 1), and multiple countries (*n* = 1). Except for one office study [43], eight studies were performed in residential buildings (Table A10). Some studies reported that energy efficiency intervention in homes can improve asthma [77,78], respiratory symptoms [77,78,79,81], sinusitis [80], general health [80,83], satisfaction [80,81], and reduce blood pressure [84]. Furthermore, an improved mechanical ventilation rate in office buildings can improve SBS symptoms, productivity, and perceived indoor air quality [43]. In addition, energy saving by reducing ventilation flow to below 0.5 air change rate (ACH) could impair perceived air quality but did not influence SBS [82].

#### 3.2.6. Energy Factors and Health

In Table 3, data on associations between energy factors and any health outcomes among all 45 selected health studies were summarized. Thermal issues, including fuel poverty or low indoor air temperature, were not included in this table. Most studies showed beneficial effects of energy saving.

## 4. Discussion

To our knowledge, this review is the first systematic review on associations between different energy aspects of buildings and health. A meta-analysis could not be performed, since there were few articles covering the same energy aspect and the same health variable. However, the current knowledge level and knowledge gaps on the health effects of green buildings, fuel poverty, and energy use as well as energy efficiency improvements in buildings was able to be summarized or described.

In this review, there were three important issues related to exposure studies. Firstly, radon concentration in six studies was above 100 Bq/m^3^ in mean or in rooms [19,20,22,24,25,27]. In one review with meta-analysis on the risk of radon, the action level of radon for never-smokers and ever-smokers was recommended at 100 Bq/m^3^ of World Health Organization. They reported that radon exposure is the strongest risk factor for lung cancer for never-smokers [86]. Thus, special concern should be taken around radon exposure when performing home energy efficiency retrofits. In order to reduce radon levels in home energy-efficiency retrofits, installation of ground covers and sub-slab or sump depressurization systems as well as mechanical ventilation could be undertaken. One main source of indoor radon is radon from the ground. It should be ensured that the transmission of radon from the ground into buildings is minimized, especially for buildings in regions with primary geological layers in the underground. Another source of indoor radon is building materials, although it is not the main source. It is highly recommended that the building material for home retrofits works should meet the standards of green buildings. Secondly, installation of insulated windows and central heating systems can increase the indoor concentrations of mould [30]. The health risk of mould had been assessed in a previous review [8]. In many countries, mould and dampness caused by critical thermal bridges is a reason why energy efficiency interventions were performed [87]. Thus, it is important to consider thermal bridges as a cause of indoor mould growth after improving insulation in buildings. Thirdly, home energy efficiency retrofits can increase benzene, toluene, ethyl benzene, and xylene (BTEX) in indoor air [29]. In one previous review, the negative health effects of indoor BTEX had been reported [88]. Thus, it is important to use low-emissions building materials in energy efficiency retrofits.

Moreover, there were four important issues related to health studies.

Firstly, there were negative health effects in buildings with thermal insulation without installation of mechanical ventilation. In most cases, thermal insulation can reduce heat transfer, which will increase indoor temperature and reduce relative humidity and risk of mould. However, since many energy efficiency improvement methods can lead to reduced ventilation rates or air tightness, special concern should be taken to compensate for the reduced natural ventilation rate when working with home energy efficiency improvements. Thus, energy efficiency methods combined with improved ventilation or design should be promoted in airtight homes. In addition, the issue of thermal bridges and mould growth was seldom mentioned in the health studies.

Secondly, there were two negative associations between improved ventilation rate and health. In a fuel poverty study, high ventilation rates in low-income urban homes may increase chronic cough, asthma, and asthma-like symptoms [54]. This could be due to increased infiltration of outdoor air pollutants. Although this knowledge may be well known, the level of outdoor air pollutants had not been evaluated by the current intervention programs of low-income homes we found. In a cross-sectional health study, buildings with balanced ventilation systems (supply/exhaust ventilation) had a higher prevalence of doctor diagnosed allergies, as compared to buildings with exhaust ventilation only [71]. This may be caused by lack of a correct replacement of dirty filters in balanced mechanical ventilation systems. Thus, this knowledge should be addressed to residents in homes with energy efficiency improvements combined with balanced ventilation systems.

Thirdly, four fuel poverty health studies on cold mortality were performed in a longitudinal study design. This means that the cold-mortality effect of fuel poverty has been well known. Thus, fuel poverty behavior should be considered in interventions since it is often linked to reduced ventilation rate and ineffective heating. Except for winter fuel payment and energy intervention policy, wearable telemetry may be a good choice of solution in cold homes [63]. This is because wearable telemetry can increase the occupant’s awareness of cold. However, all those studies were based on cold climates. In hot climate zones, there is a need to conduct similar research in low-income homes.

Fourthly, 4 green buildings health studies were conducted in a longitudinal study design. This means that long-term health effects of green buildings were assessed in the USA. However, those green buildings were assessed by LEED credits of the USA standard. Although there are existing green rating systems in different countries, energy efficiency improvements combined with correct ventilation and renewable energy use have been emphasized in most green rating systems.

This literature review has a number of strengths. The main focus was on epidemiological studies, including intervention studies, cross-sectional studies, and longitudinal studies. However, exposure studies without any reported health data or health associations were also included if they were identified in this literature search. For each included study, the country of the study, type of study, type of buildings, number of buildings and number of subjects were noted in the review. In exposure studies, extra information on the changes of concentrations of major pollutants was collected. In studies with unexpected results or negative impacts of energy use and energy saving, explanations of the results reported by the authors were included.

The studies included in this review had some limitations in their study design. One major limitation was that none of the studies had studied health effects of energy efficiency improvement by the installation of heat recovery to existing mechanical ventilation systems. This may be because many studies had not separated it from combined energy efficiency measures. However, installation of heat recovery to mechanical ventilation systems is a major method nowadays to save energy use and there is a need to assess its health benefits, especially in airtight homes. The second limitation is that many of the intervention studies were based on more than two energy saving improvements. Thus, it is not possible to draw clear conclusions on the health effects of single energy efficiency improvement measures. The third limitation is that there were few prospective health studies on long-term health effects of energy efficiency improvements and energy use. However, many prospective health studies on green buildings and fuel poverty were found. The fourth limitation is that most studies were on residential buildings. Only three studies were on office buildings and only four studies were on school or university buildings.

## 5. Conclusions

Energy efficiency improvements and green building can have positive effects on asthma, respiratory symptoms, mental health, and general health as well as on performance and satisfaction. Home energy efficiency improvement with mechanical ventilation system can reduce radon, mould, bacteria, and house dust mites, TVOC, CO_2_, CO, and black carbon levels as well as some volatile organic compounds. More efficient heating and increased thermal insulation can have positive health impacts in fuel-poverty homes. However, energy savings in airtight buildings and thermal insulation without the installation of mechanical ventilation can impair health. Moreover, health risks linked to energy efficiency retrofits exists. Installation of mechanical ventilation can solve many of the negative effects linked to airtight buildings and energy efficiency retrofits.

For future energy efficiency intervention or retrofit studies, measures of radon and BTEX and other chemicals, as well as levels of thermal bridge and outdoor air pollutants may be needed. In addition, it is important to replace dirty filters in balanced mechanical ventilation systems.

Furthermore, future research needs on this topic were identified. Firstly, the intervention study should measure how much energy they save after energy efficiency measures. Secondly, more studies are needed on the health aspects of energy efficiency improvement by the installation of heat recovery to mechanical ventilation system. Thirdly, future studies should focus on evaluating health effects of single energy efficiency improvement measures, rather than a combination of measures. Fourthly, more prospective health studies on long-term health effects of energy efficiency improvements or energy use are needed. Fifthly, future studies should include offices, schools, and hospital buildings, and should cover different climate zones in the world.

## Figures and Tables

**Table 1:** Associations between energy-related building factors and pollutants among the 23 included exposure studies.

No.	References	Country	Pollutant Groups	Improved Ventilation	Thermal Retrofit	Draught Proofing	Green Retrofits	Fuel Poverty	Energy Carrier
1	Collignan et al. 2016 [19]	France	Radon						
2	Symonds et al. 2019 [20]	United Kingdom	Radon		↑				
3	Meyer et al. 2019 [21]	Germany	Radon		↑	↑			
4	Pressyanov et al. 2015 [22]	Bulgaria	Radon		↑	↑			
5	Vasilyev et al. 2017 [23]	Russia	Radon		↑				
6	Yarmoshenko et al. 2014 [24]	Russia	Radon		↑				
7	Vasilyev et al. 2015 [25]	Russia	Radon		↑				
8	Burghele et al. 2020 [26]	Romania	Radon	↓					
9	Pigg et al. 2018 [27]	United States	Radon ^1^, Chemicals ^3^	↓	↑^1^, ↓^3^	↑^1^, ↓^3^			
10	Wallner et al. 2015 [28]	Austria	Radon ^1^, Biological agents ^2^, Chemicals ^3^	↓^1^, ↓^2^, ↓^3^					
11	Du et al. 2019 [29]	Finland Lithuania	Radon ^1^, Biological agents ^2^, Chemicals^3^	↓^2^, ↑↓^3^	↑^1^				
12	Hirsch et al. 2020 [30]	Germany	biological agents		↑				
13	Sharpe et al. 2015 [31]	United Kingdom	biological agents					↑	
14	Sharpe et al. 2016 [32]	United Kingdom	biological agents	↓					
15	Spertini et al. 2010 [33]	Switzerland	biological agents	↓					
16	Niculita-Hirzel et al. 2000 [34]	Switzerland	biological agents	↓					
17	Coombs et al. 2018 [35]	United States	biological agents				0		
18	Derbez et al. 2018 [36]	France	Chemicals		↑				
19	Leivo et al. 2018 [37]	Finland Lithuania	Chemicals	↓	↑				
20	Coombs et al. 2016 [38]	United States	Chemicals				↑↓		
21	Yang et al. 2020 [39]	Switzerland	Chemicals		↑				
22	Verriele et al. 2016 [18]	France	Chemicals	↓					
23	Baumgartner et al. 2019 [40]	China	Chemicals						↑↓

↑ means increase, ↓ means decrease, ↑↓ means mixed results, 0 means no associations. ^1^ represents radon, ^2^ represents biological agents, ^3^ represents chemicals.

**Table 2:** Associations between one kind of fuel poverty, improved ventilation, and energy efficiency improvements and health in all 45 selected health studies.

No.	Reference	Thematic Group	Respiratory Health		General Health	Mental Health	Performance	Satisfaction	Cold-Related Mortality	SBS Symptoms
			Asthma	Other Respiratory Illnesses						
1	Garland et al. 2013 [47]	Green Buildings	+							
2	Singh et al. 2010 [41]	Green Buildings	+	+		+	+			
3	Breysse et al. 2011 [48]	Green Buildings	+	+	+					
4	Breysse et al. 2015 [49]	Green Buildings			+	+				
5	Hedge et al. 2013 [44]	Green Buildings			+		+	+		
6	Hedge et al. 2014 [45]	Green Buildings			+		+	+		
7	Gawande et al. 2020 [42]	Green Buildings								0
8	Rudge et al. 2005 [50]	Fuel Poverty	#							
9	Webb et al. 2013 [51]	Fuel Poverty	#							
10	Sharpe et al. 2015 [52]	Fuel Poverty	#							
11	Poortinga et al. 2017 [53]	Fuel Poverty	+		+/−	+		+/−		
12	Carlton et al. 2019 [54]	Fuel Poverty	−	−						
13	Howden-Chapman et al. 2011 [55]	Fuel Poverty	+		+	+				
14	Howden-Chapman et al. 2007 [56]	Fuel Poverty	+		+					
15	Humphrey et al. 2020 [54]	Fuel Poverty		+						
16	Thomson et al. 2017 [57]	Fuel Poverty			#	#				
17	Ahrentzen et al. 2016 [58]	Fuel Poverty			+	+				
18	Shortt et al. 2007 [59]	Fuel Poverty			+	+				
19	Chapman et al. 2009 [60]	Fuel Poverty			+					
20	Grey et al. 2017 [61]	Fuel Poverty				+		+		
21	Poortinga et al. 2018 [62]	Fuel Poverty				+		+		
22	Pollard et al. 2019 [63]	Fuel Poverty						#		
23	Angelini et al. 2019 [64]	Fuel Poverty							#	
24	Sartini et al. 2018 [65]	Fuel Poverty							+	
25	Peralta et al. 2017 [66]	Fuel Poverty							+/−	
26	Umishio et al. 2019 [67]	Fuel Poverty							#	
27	López-Bueno et al. 2020 [68]	Fuel Poverty							+	
28	Engvall et al. 2003 [69]	Cross sectional								−
29	Smedje et al. 2017 [70]	Cross sectional								+
30	Norback et al. 2014 [71]	Cross sectional		+/−						
31	Wang et al. 2017 [72]	Cross sectional	+							
32	Sharpe et al. 2019 [73]	Cross sectional	+							
33	Sobottka et al. 1996 [74]	Cross sectional								−
34	Bakke et al. 2008 [46]	Cross sectional			#					
35	Kennard et al. 2020 [75]	Cross sectional			#					
36	Wallner et al. 2017 [76]	Longitudinal			+					
37	Somerville et al. 2000 [77]	Intervention	+	+						
38	Barton et al. 2007 [78]	Intervention	+	+						
39	Osman et al. 2010 [79]	Intervention	+							
40	Wilson et al. 2013 [80]	Intervention		+	+			+		
41	Haverinen-Shaughnessy et al. 2018 [81]	Intervention	+					+		
42	Wargocki et al. 2000 [43]	Intervention					+			+
43	Engvall et al. 2005 [82]	Intervention								0
44	Francisco et al. 2017 [83]	Intervention			+					
45	Umishio et al. 2020 [84]	Intervention							+	

Remark: + mean positive result, − means negative result, +/− means mixed results, 0 means no associations, # means fuel poverty issues. SBS: sick building syndrome.

**Table 3:** Associations between energy factors and any health outcomes among all 45 selected health studies.

No.	References	Energy Efficiency Improvements (at Least Two Measures)	Green Buildings	More Effective Heating	Thermal Insulation	Draught Proofing	Higher Ventilation Rate	Installation of Mechanical Ventilation
1	Garland et al. 2013 [47]		+					
2	Singh et al. 2010 [41]		+					
3	Breysse et al. 2011 [48]		+					
4	Breysse et al. 2015 [49]		+					
5	Hedge et al. 2013 [44]		+					
6	Hedge et al. 2014 [45]		+					
7	Gawande et al. 2020 [42]		+					
8	Poortinga et al. 2017 [61]	+			−			
9	Carlton et al. 2019 [54]						−	
10	Howden-Chapman et al. 2011 [55]			+	+			
11	Howden-Chapman et al. 2007 [56]				+			
12	Humphrey et al. 2020 [85]						+	
13	Ahrentzen et al. 2016 [58]	+						
14	Shortt et al. 2007 [59]			+	+			
15	Chapman et al. 2009 [60]				+			
16	Grey et al. 2017 [61]	+						
17	Poortinga et al. 2018 [62]	+						
18	Sartini et al. 2018 [65]				+			
19	Peralta et al. 2017 [66]				+/−			
20	López-Bueno et al. 2020 [68]			+				
21	Engvall et al. 2003 [69]			+		−		+
22	Smedje et al. 2017 [70]				+			
23	Norback et al. 2014 [71]			+				
24	Wang et al. 2017 [72]						+	
25	Sharpe et al. 2019 [63]					−		
26	Sobottka et al. 1996 [74]					−		
27	Wallner et al. 2017 [76]	+						
28	Somerville et al. 2000 [77]			+				
29	Barton et al. 2007 [78]	+						
30	Osman et al. 2010 [79]	+						
31	Wilson et al. 2013 [80]	+						
32	Haverinen-Shaughnessy et al. 2018 [81]				+			
33	Wargocki et al. 2000 [43]						+	
34	Engvall et al. 2005 [82]						+	
35	Francisco et al. 2017 [83]				+			
36	Umishio et al. 2020 [84]				+			
Positive associations (+)		8	7	6	9		4	1
Negative associations (−)					1	3	1	
Mixed results (+/−)					1			

Remark: + mean positive result, − means negative result, +/− means mixed results.

## Appendix A

**Table A1:** Exposure Field Studies on Radon.

Author	Year	Country	Energy Aspects	Type of Study	Type of Buildings	Measured Exposure	Changes of Measured Exposure	Number of Households or Buildings	Main Results
Collignan et al. [19]	2016	France	Improved insulation, ventilation and window replacement	One time measurement	Residential buildings	Radon	21% increase (median 147 Bq/m^3^)	3233 households or 3233 buildings	Energy efficiency thermal retrofit (linked to reduced air permeability of the building envelope) can increase indoor radon concentration.
Symonds et al. [20]	2019	United Kingdom	Insulation in loft and wall, double glazing	Longitudinal	Residential buildings	Radon	Arith. Mean > 132–159.3 Bq/m^3−^	470,689 households	Energy efficiency retrofit by improving insulation in loft and wall, and/or double glazing can increase radon concentrations, possibly due to increased airtightness.
Du et al. [29]	2019	Finland and Lithuania	Improved insulation in wall, roof, windows or balconies	Intervention	Residential buildings	Radon	Mean increase of 13.8 Bq/m^3^ (<100 Bq/m^3^)	336 households or 65 buildings	In homes in Lithuania, energy efficiency retrofits without installation of mechanical ventilation increased indoor radon concentrations.
Pigg et al. [27]	2018	United States	Weatherization services	Intervention	Residential buildings	Radon	Increased by 0.14 ± 0.13 pCi/L (>100 Bq/m^3^ in high zone)	514 households	Energy efficiency weatherization services (retrofits) can increase radon concentration. However, energy efficiency weatherization services with improved ventilation or ground covers can reduce radon concentration.
Meyer et al. [21]	2019	Germany	Air tightness windows and insulation of outer walls	One time measurement	Residential buildings	Radon	40 Bq/m^3^ in non-refurbished vs. 69 Bq/m^3^ in refurbished	150 households	Energy efficiency refurbishments of existing buildings without installation of ventilation systems can increase radon concentration, as compared to non-refurbished conventional buildings.
Pressyanov et al. [22]	2015	Bulgaria	New energy-efficient windows with plastic joinery	Intervention	Residential buildings	Radon	Rooms with radon increase was 193 Bq/m^3^ and rooms with no change was 45 Bq/m^3^	20 rooms or 16 buildings	Energy-efficient reconstructions with installation of new energy-efficient windows (linked to air tightness) can increase radon levels.
Vasilyev et al. [23]	2017	Russia	Energy efficiency insulation	One time measurement	Residential buildings	Radon	Arithmetic mean 38 Bq/m^3^ in conventional vs. 93 Bq/m^3^ in modern buildings	81 buildings	Energy efficiency measures in buildings (linked to low indoor air exchange rate) can increase indoor radon concentration.
Yarmoshenko et al. [24]	2014	Russia	Energy efficiency insulation	Before-after	Residential buildings	Radon	Arithmetic mean 42 Bq/m^3^ in conventional vs. 133 Bq/m^3^ in modern buildings	7 households or 7 buildings	Energy efficiency measures in buildings (linked to low indoor air exchange rate) can increase indoor radon concentration.
Vasilyev et al. [25]	2015	Russia	Energy efficiency insulation	Before-after	Residential buildings	Radon	Arithmetic mean 42 Bq/m^3^ in conventional vs. 166 Bq/m^3^ in modern buildings	5 rooms or 5 buildings	Energy efficiency measures in buildings (linked to low indoor air exchange rate) can increase indoor radon concentration.
Burghele et al. [26]	2020	Romania	Installation of centralized and decentralized mechanical ventilation with heat recovery	Intervention	Residential buildings	Radon	Reduction was between 25% to 95% (Before >100 Bq/m^3^)	10 households or 10 buildings	Sub-slab and sump depressurization, installation of centralized and decentralized mechanical ventilation with heat recovery can reduce radon concentrations.
Wallner et al. [28]	2015	Austria	Existing mechanical ventilation and natural ventilation	Before-after	Residential buildings	Radon	17 Bq/m^3^ mechanical ventilation vs. 31 Bq/m^3^ natural ventilation	123 households	Energy-efficient buildings with existing mechanical ventilation can reduce radon concentrations, as compared to conventional buildings without installation of mechanical ventilation, especially for radon.

**Table A2:** Exposure Field Studies on Mould Bacteria and House Dust Mites.

Author	Year	Country	Energy Aspects	Type of Study	Type of Buildings	Measured Exposure	Changes of Measured Exposure	Number of Households	Main Results
Hirsch et al. [30]	2000	Germany	Installation of insulated windows and central heating systems	Intervention	Residential buildings	House dust mite Der f 1 and mould	Der f 1 in carpets 0.65 vs. 1.28, mattresses 1.56 vs. 2.40 μg/g; Aspergillus fumigatus 20 vs. 60 units/g	98 households	Installation of insulated windows and central heating systems (linked to reduced ventilation) increased the concentration of the house dust mite allergen Der f 1 and the mould species Aspergillus fumigatus.
Sharpe et al. [31]	2015	United Kingdom	Fuel poverty	Cross-sectional	Social Residential buildings	Self-reported dampness and mould	No data	671 households	Fuel poverty (linked to ineffective heating and ventilation practices) can increase indoor dampness and mould, regardless of the use of extractor fans.
Sharpe et al. [32]	2016	United Kingdom	Type of heating, glazing, insulation levels, energy efficiency ratings	Cross-sectional	Social Residential buildings	Self-reported allergenic mould	No data	41 households	Energy efficiency improvement combined with increased ventilation flow rate reduced fungal contamination with Aspergillus/Penicillium mould species and *Cladosporium* spp.
Spertini et al. [33]	2010	Switzerland	Improved insulation, ventilation system with heat recovery and natural ventilation	One time measurement	Residential buildings	Self-reported house dust mites Der f 1	Median 67 vs. 954 ng/g in mattresses and 20 vs. 174 ng/g in carpets	289 households or 11 buildings	Buildings designed for low energy use with installation of mechanical ventilation reduced indoor relative air humidity as well as house dust mite allergen concentration both in mattresses and in carpets, as compared to control buildings.
Niculita-Hirzel et al. [34]	2020	Switzerland	Type of ventilation and energy consumption	One time measurement	Residential buildings	Fungal	Penicillium CFUs was lower	149 households	Installation of mechanical ventilation in buildings reduced the infiltration of outdoor fungal particles, as compared to buildings with natural ventilation only.
Coombs et al. [35]	2018	United States	Green renovation with bathroom fans	Before-after	Residential buildings	Mould	521,826 reads from green homes vs. 726,690 fungal reads from non-green homes	52 households	The concentration of mould in air samples and door dust samples did not differ between green and non-green homes. However, green homes had a lower concentration of mould in bed samples.
Du et al. [29]	2019	Finland and Lithuania	Replacing windows and/or installation of heat recovery to the existing exhaust ventilation system	Intervention	Residential buildings	Airborne mould and bacterial	Fungal 0.6-log; Bacterial 0.6-log in gram-positive and 0.9-log in gram-negative bacterial (reduction in cells/m^2^)	336 households or 65 buildings	In homes in Finland, energy efficiency retrofits with installation of mechanical ventilation reduced indoor concentrations of airborne mould and bacterial.
Wallner et al. [28]	2015	Austria	Mechanical ventilation and natural ventilation	Before-after	Residential buildings	Mould	84% of rooms vs. 35% rooms	123 households	Energy-efficient buildings with installation of mechanical ventilation reduced indoor mold spore concentration, as compared to conventional buildings without installation of mechanical ventilation.

**Table A3:** Exposure Field Studies on Chemicals.

Author	Year	Country	Energy Aspects	Type of Study	Type of Buildings	Measured Exposure	Changes of Measured Exposure	Number of Households or Buildings	Main Results
Derbez et al. [36]	2018	France	Installation of ventilation system or passive stack/hybrid ventilation	Before-after	Residential buildings	VOCs, aldehyde	Hexaldehyde: 37 vs. 17 µg/m^3^ in dwellings with/without flooring products	72 households or 43 buildings	Low energy retrofit can increase the air concentration of alpha-pinene and hexaldehyde, possibly caused by the use of wood or wood-based products for the construction and insulation.
Du et al. [29]	2019	Finland and Lithuania	Replacing windows and/or installation of heat recovery to the existing exhaust ventilation system	Intervention	Residential buildings	BTEX and formaldehyde	Mean increase of 2.5 µg/m^3^ in BTEX	336 households or 65 buildings	In homes in Finland, energy efficiency retrofits with existing mechanical ventilation increased indoor air concentrations of benzene, toluene, ethyl benzene and xylene (BTEX) but reduced indoor formaldehyde concentrations.
Leivo et al. [37]	2018	Finland and Lithuania	Installation of heat recovery to the existing exhaust ventilation system. Improved thermal insulation in wall, roof, windows or balconies	Intervention	Residential buildings	CO_2_	Median: 775 vs. 956 PPM (1st); Median: 730 vs. 840 PPM (2nd)	290 households or 66 buildings	In homes in Finland, energy efficiency retrofits with existing mechanical ventilation reduced CO_2_ concentration as compared to natural ventilation. In homes in Lithuania, improved insulation without installation of mechanical ventilation increased measured CO_2_ levels.
Coombs et al. [38]	2016	United States	Green renovation	Intervention	Residential buildings	Black carbon, formaldehyde	Black carbon averaging 682 vs. 2364 ng/m^3^; Formaldehyde 0.03 vs. 0.01 ppm	42 households	Energy efficiency green renovation (linked to reduced ventilation) decreased indoor black carbon level in air from outdoor sources and increased indoor formaldehyde concentration.
Yang et al. [39]	2020	Switzerland	Thermal retrofit of roof, walls and floors, replacement of heating system, installation of mechanical ventilation system	One time measurement	Residential buildings	Aldehydes, VOCs	Formaldehyde 13 vs. 15; Toluene 16 vs. 26; Xylenes 1.4 vs. 5.8; Acrolein 0.4 vs. 0.6; D-limonene 7.9 vs. 11; Isobutane 3.4 vs. 10; Butane 8.8 vs. 2 (µg/m^3^)	169 households	Energy efficiency thermal retrofit without installation of mechanical ventilation increased formaldehyde, aromatics, alkane, and levels of certain volatile organic compounds, as compared to new homes built with installed mechanical ventilation.
Verriele et al. [18]	2016	France	Controlled ventilation systems	Before-after	School buildings	CO_2_	Peak Level 1000 ppm vs. 3800–5000 ppm	10 school buildings	Low energy school buildings combined controlled mechanical ventilation systems and an adapted ventilation schedule can reduce CO_2_ levels.
Pigg et al. [27]	2018	United States	Weatherization services	Intervention	Residential buildings	CO	Peak Level 35 ppm vs. 13–20 ppm	514 households	Energy efficiency weatherization services in homes without improved ventilation or ground covers can reduce exposure to CO.
Wallner et al. [28]	2015	Austria	Mechanical ventilation and natural ventilation	Before-after	Residential buildings	CO_2_, TVOC, aldehydes	CO_2_: 1360 vs. 1830 ppm and 1280 vs. 1740 ppm; TVOC: 300 vs. 560 µg/m^3^; aldehydes: 32 vs. 53 µg/m^3^ and 18 vs. 33 µg/m^3^	123 households	Energy efficient buildings with installation of mechanical ventilation reduced indoor concentrations of CO_2_, TVOC, aldehydes, and improved the measured indoor air quality in homes, as compared to conventional buildings without installation of mechanical ventilation.
Baumgartner et al. [40]	2019	China	Low-polluting semi gasifier cook stove with chimney, water heater and pelletized biomass fuel	Intervention	Residential buildings	PM_2.5_, black carbon	PM_2.5_ (46%), black carbon (55%)	205 households	An energy intervention replacing low-polluting semi gasifier cook stove in rural buildings was associated with decreased exposures to PM_2.5_ and black carbon in winter but higher exposures to PM_2.5_ and black carbon in summer, as compared to untreated homes with traditional stoves. The negative effect could be caused by increased use of semi gasifier cook stove.

**Table A4:** Green Building Health Studies.

Author	Year	Country	Energy Aspects	Type of Study	Type of Buildings	Type of Health Variables	Number of Buildings	Number of Subjects	Main Results
Garland et al. [47]	2013	United States	Green buildings with LEED Credits	Intervention	Residential buildings	Self-reported asthma	1 building	18 children and adults	Green home buildings can reduce self-reported asthma symptoms.
Singh et al. [41]	2010	United States	Green buildings with LEED Credits	Longitudinal	Office buildings	Absenteeism due to self-reported asthma, respiratory allergies, depression and stress, and work productivity	2 office buildings	263 employees	Green office buildings can improve indoor environment quality and reduce absenteeism due to self-reported asthma, respiratory allergies, depression and stress, and moreover improve work productivity.
Breysse et al. [48]	2011	United States	Green efficiency renovation	Longitudinal	Residential buildings	Self-reported asthma, and non-asthma respiratory problems, overall health	1 building	80 children and adults	Among adults, green efficiency renovation in homes can reduce self-reported asthma. Green efficiency renovation in homes can improve self-reported overall health and reduce non-asthmatic respiratory symptoms in adults as well as in children.
Breysse et al. [49]	2015	United States	Green efficiency renovation	Intervention	Residential buildings	Self-reported mental and general physical health	1 building	612 older adults	Green efficiency renovation in homes can improve mental and general physical health.
Hedge et al. [44]	2013	United States	Green buildings with LEED Credits	Longitudinal	University buildings	Overall health, performance and work satisfaction	2 university buildings	44 employees	Green office buildings in a college campus improved health, performance and work satisfaction.
Hedge et al. [45]	2014	Canada	Green buildings with LEED Credits	Longitudinal	University buildings	Overall health, performance and study satisfaction	3 university buildings	319 employees	Green classrooms in an university campus improved health, performance and work satisfaction.
Gawande et al. [42]	2020	India	Green Office Buildings	cross-sectional	Office Buildings	SBS	10 office buildings	148 employees	No significant association between green buildings and sick building syndrome symptoms (SBS), compared with conventional buildings.

**Table A5:** Fuel Poverty Health Studies on Respiratory Symptoms.

Author	Year	Country	Energy Aspects	Type of Study	Type of Buildings	Type of Health Variables	Number of Households	Number of Subjects	Main Results
Rudge et al. [50]	2005	United Kingdom	Fuel poverty	Longitudinal	Residential buildings	Winter respiratory disease	220 households	460 older adults	Fuel poverty in low-income homes can increase winter respiratory disease.
Webb et al. [51]	2013	United Kingdom	Fuel poverty	Cross-sectional	Residential buildings	Measured respiratory disease	3763 households	3763 older adults	Fuel poverty in low-income homes increase respiratory disease.
Sharpe et al. [52]	2015	United Kingdom	Type of heating, glazing, insulation, energy efficiency ratings	Cross-sectional	Social residential buildings	Doctor diagnosed asthma	706 households	944 adults	Energy efficiency improvement in social housing might increase current adult asthma. This may be due to increased exposure to physical, biological and chemical contaminants linked to inadequate heating, ventilation (fuel poverty behavior).
Poortinga et al. [53]	2017	United Kingdom	Loft insulation, cavity-wall insulation, external wall insulation	Repeated cross-sectional	Social residential buildings	General health, mental health, and social outcomes	Around 9200 households	10,009 individuals	Energy efficiency improvements in social housing can improve respiratory symptoms.
Carlton et al. [54]	2019	United States	Home ventilation rate	Cross-sectional	Residential buildings	Respiratory symptoms	216 households	302 children and adults	High ventilation rates in low-income urban homes may increase chronic cough, asthma and asthma-like symptoms, probably caused by infiltration of outdoor air pollutants.
Howden-Chapman et al. [55]	2011	New Zealand	Improved insulation into existing houses; more effective heating in insulated houses	Intervention	Residential buildings	Respiratory symptoms	1350 households; 409 households	4407 children and adults; 409 children	Energy saving by using more effective heating in insulated low-income homes can improve health status and respiratory symptoms in children with asthma diagnosis.
Howden-Chapman et al. [56]	2007	New Zealand	Installation of a standard retrofit insulation package	Intervention	Residential buildings	Hospital admissions for respiratory conditions	1350 households	4407 children and adults	Energy saving by insulating existing houses in low-income communities can improve indoor environment and reduce hospital admissions for respiratory conditions.
Humphrey et al. [85]	2020	United States	Home ventilation rate	Cross-sectional	Residential buildings	Measured lung function	187 households	253 children and adults	High infiltration rate in low-income, urban, non-smoking homes can improve lung health.

**Table A6:** Fuel Poverty Health Studies on General and Mental Health.

Author	Year	Country	Energy Aspects	Type of Study	Type of Buildings	Type of Health Variables	Number of Households	Number of Subjects	Main Results
Thomson et al. [57]	2017	32 European Countries	Fuel poverty	Cross-sectional	Residential buildings	General health and well-being	No data	41,560 adults	Fuel poverty in low-income homes can reduce general health and emotional well-being.
Poortinga et al. [53]	2017	United Kingdom	Loft insulation, cavity-wall insulation, external wall insulation	Repeated cross-sectional	Social residential buildings	General health, mental health, and social outcomes	Around 9200 households	10,009 individuals	Energy efficiency improvements in social housing can improve general health, mental health and social outcomes. However, installation of cavity wall insulation without installation of mechanical ventilation can reduce general health outcomes and social outcomes.
Ahrentzen et al. [58]	2016	United States	Insulation of roof and floor, improved thermal, air conditioner heating, cooling system, new ceiling fans, new windows	Intervention	Residential buildings	General health, emotional distress, sleep	53 households	57 older adults	Energy efficiency retrofits in low-income homes can improve general health, emotional distress, and sleep among the older adults.
Shortt et al. [59]	2007	United Kingdom	Installation of central heating systems or improved insulation	Intervention	Residential buildings	General health, well being	100 households	100 individuals	Energy efficiency intervention in fuel poverty homes can improve general health, well-being.
Howden-Chapman et al. [55]	2011	New Zealand	Improved insulation into existing houses; more effective heating in insulated houses	Intervention	Residential buildings	General health and well being	1350 households; 409 households	4407 children and adults; 409 children	Energy saving by improving insulation in low-income homes can improve general health and well-being and reduce hospitalization in children and adults. Energy saving by using more effective heating in insulated low-income homes can improve general health status in children.
Howden-Chapman et al. [56]	2007	New Zealand	Installation of a standard retrofit insulation package	Intervention	Residential buildings	General health and well being	1350 households	4407 children and adults	Energy saving by insulating existing houses in low-income communities can improve indoor environment so that improve self-reported health, wheezing, days off school and work.
Chapman et al. [60]	2009	New Zealand	Installation of a standard retrofit insulation package	Intervention	Residential buildings	General health, well being	1350 households	4407 children and adults	Energy saving by insulating existing houses in low-income communities can improve general health, as well as cost-benefit of general practitioner (GP) visits, hospitalizations, reduced time off work and school.
Grey et al. [61]	2017	United Kingdom	External wall insulation, central heating system, and installation of gas network	Intervention	Residential buildings	Well-being and psychosocial outcomes	774 households	776 individuals	Energy efficiency intervention in low-income homes can increase residential wellbeing and psychosocial-related health.
Poortinga et al. [62]	2018	United Kingdom	External wall insulation, photovoltaics, solar water heating, air source heat pumps, loft/rafter insulation	Intervention	Residential buildings	Well-being and psychosocial outcomes	4968 households	25,908 individuals	Energy efficiency intervention in low-income homes can improve well-being and psychosocial outcomes.
Pollard et al. [63]	2019	United Kingdom	Fuel poverty	Intervention	Residential buildings	Psychosocial outcomes	22 households	22 adults	Wearable telemetry (a thermometer with a low-temperature alarm) can raise awareness of the health effects of cold home among people living in fuel poverty (linked to psychosocial outcomes).

**Table A7:** Fuel Poverty Health Studies on Cold-related Mortality.

Author	Year	Country	Energy Aspects	Type of Study	Type of Buildings	Type of Health Variables	Number of Households	Number of Subjects	Main Results
Angelini et al. [64]	2019	United Kingdom	Winter fuel payment	Longitudinal	Residential buildings	Cold-related mortality, blood pressure, fibrinogen	11,578 households	18,813 adults	Low indoor air temperature in low-income homes can increase systolic and diastolic blood pressure and fibrinogen levels in blood samples (linked to cold-related mortality).
Sartini et al. [65]	2018	United Kingdom	Fuel poverty, types of home insulation and heating	Longitudinal	Residential buildings	Cold-related mortality	1006 households	1402 older men	Lack of insulation in low-income homes can increase cold-related mortality.
Peralta et al. [66]	2017	Spain	Energy efficient façade insulation retrofit	Longitudinal	Social residential buildings	Cold-related mortality	2552 households	2552 individuals	Energy efficient façade insulation retrofit in public housing can reduce cold-related mortality in women, but can increase cold-related total mortality in men. The health outcome for the gender difference is unclear.
Umishio et al. [67]	2019	Japan	Low insulation in cold homes	Cross-sectional	Residential buildings	Blood pressure	1840 households	2900 adults	Low indoor air temperature was higher associated with blood pressure and hypertension (linked to cold-related mortality).
López-Bueno et al. [68]	2020	Spain	Heating systems	Longitudinal	Residential buildings	Cold-related mortality	No data	No data	Districts with higher homes without a heating system had cold-related mortality.

**Table A8:** Cross-sectional Health Studies.

Author	Year	Country	Energy Aspects	Type of Study	Type of Buildings	Type of Health Variables	Number of Buildings or Households	Number of Subjects	Main Results
Engvall et al. [69]	2003	Sweden	Type of ventilation and heating system, heat pumps, reconstruction and energy-saving measures	Cross-sectional	Residential buildings	Sick building syndrome (SBS) symptoms	231 buildings	3241 adults	In multi-family buildings, lack of a mechanical ventilation system and use of direct electric radiators were associated with increased prevalence of SBS-related symptoms. Major reconstruction and multiple sealing in multi-family buildings were associated with increased prevalence of SBS-related symptoms.
Smedje et al. [70]	2017	Sweden	Type of ventilation system and insulation level	Cross-sectional	Residential buildings	Sick building syndrome (SBS) symptoms	605 buildings	1160 adults	In single-family buildings, a lower U-value (higher insulation level) was associated with less SBS symptoms.
Norback et al. [71]	2014	Sweden	Type of ventilation and energy use for heating	Cross-sectional	Residential buildings	Doctor’s diagnosed asthma, allergy and self-reported pollen allergy, eczema	472 buildings	7554 adult	Multi-family buildings with balanced ventilation systems (supply/exhaust ventilation) had a higher prevalence of doctor diagnosed allergy, as compared to buildings with exhaust ventilation only. Buildings using more energy for heating were associated with less pollen allergy and eczema.
Wang et al. [72]	2017	Sweden	Type of ventilation and degree of insulation	Cross-sectional	Residential buildings	Doctors’ diagnosed asthma, self-reported asthma	605 buildings	1160 adults	Higher air exchange rate in the single-family residential homes was associated with less current asthma symptoms.
Sharpe et al. [73]	2019	United Kingdom	Energy efficiency ratings	Cross-sectional	Residential buildings	Asthma, chronic obstructive pulmonary disease, cardiovascular disease	No data	No data	Reduced home ventilation rates were associated with more asthma disease. Homes with more energy efficiency improvements may were associated with more admission rates for respiratory and cardiovascular diseases, possibly caused by reduced home ventilation flow rate. Energy efficiency measures can improve health outcomes, especially chronic respiratory illness.
Sobottka et al. [74]	1996	Germany	New windows and door, improved insulation and heating system	Cross-sectional	Residential buildings	Sick building syndrome (SBS) symptoms	52 buildings	No data	Energy saving by installing new air tightness windows and door in homes was associated with more SBS-related health complaints. This may be due to fuel poverty behavior by not airing their flats sufficiently.
Bakke et al. [46]	2008	Norway	Lower air temperature	Cross-sectional	University buildings	Tear film stability, nasal patency	4 university buildings	173 employees	Lower air temperature in buildings at a university campus was associated with less tear film stability and more health problems.
Kennard et al. [75]	2020	United Kingdom	Space heating energy use	Cross-sectional	Residential buildings	Cold-mortality	77,762 households	77,762 adults	Higher thermal variety (linked to lower domestic demand temperatures) was associated with less morbidities related to cold-mortality.

**Table A9:** Longitudinal Health Studies.

Author	Year	Country	Energy Aspects	Type of Study	Type of Buildings	Type of Health Variables	Number of Buildings	Number of Subjects	Main Results
Wallner et al. [76]	2017	Austria	Mechanical ventilation and natural ventilation	Longitudinal	Residential buildings	General health and dry eye symptoms	123 buildings	575 children and adults	Energy efficient buildings combined with installation of mechanical ventilation can improve self-reported health but increase dry eye symptoms, as compared to conventional buildings with natural ventilation only.

**Table A10:** Intervention Health Studies.

Author	Year	Country	Energy Aspects	Type of Study	Type of Buildings	Type of Health Variables	Number of Buildings or Households	Number of Subjects	Main Results
Somerville et al. [77]	2000	United Kingdom	the installation of central heating systems	Intervention	Residential buildings	Respiratory symptoms	59 households	72 children with diagnosed asthma	Energy efficiency intervention in homes reduced respiratory symptoms, and reduced missed school days due to asthma in children with diagnosed asthma.
Barton et al. [78]	2007	United Kingdom	Central heating systems, ventilation, rewiring, insulation, and re-roofing	Intervention	Social residential buildings	Asthma, non-asthma-related respiratory disease	119 households	480 children and adults	Energy efficiency intervention in social housing reduced asthma symptoms, and non-asthmatic respiratory disease.
Osman et al. [79]	2010	United Kingdom	Central heating systems, installation of loft, under-floor and cavity wall insulation	Intervention	Residential buildings	Diagnosed chronic obstructive pulmonary disease (COPD)	178 households	178 older adults with COPD	Energy efficiency intervention in homes can improve respiratory health for elderly COPD patients.
Wilson et al. [80]	2013	United States	Insulation, heating equipment and ventilation improvements	Intervention	Residential buildings	Sinusitis, general health, satisfaction	248 households	323 children and adults	Energy efficiency retrofits work in homes can improve sinusitis, general health, satisfaction.
Haverinen-Shaughnessy et al. [81]	2018	Finland and Lithuania	Installation of heat recovery to the existing exhaust ventilation system. Improved thermal insulation in wall, roof, windows or balconies	Intervention	Residential buildings	Respiratory symptoms	66 buildings	283 individuals	Energy efficiency retrofits in homes can improve occupant satisfaction with daily noise nuisance, upper respiratory symptoms, and reduce absence from school or from work due to respiratory infections.
Wargocki et al. [43]	2000	Denmark	different ventilation rates	Intervention	Office building	Sick building syndrome (SBS) symptoms, work productivity	1 office building	30 female employees	Improved mechanical ventilation rate in office building improve SBS symptoms, work productivity, and perceived indoor air quality.
Engvall et al. [82]	2005	Sweden	different ventilation rates	Intervention	Residential buildings	General health	1 building	44 adults	Energy saving by reducing ventilation flow to below 0.5 ACH could impair perceived air quality but did not influence SBS.
Francisco et al. [83]	2017	United States	Weatherization services	Intervention	Residential buildings	General health	72 households	178 children and adults	Energy efficiency retrofits can improve self-reported health.
Umishio et al. [84]	2020	Japan	installation of outer walls, floor and/or roof insulation and replacement of windows	Intervention	Residential buildings	Blood pressure	1009 households	1685 adults	Energy efficiency insulation retrofitting in homes can reduce home blood pressure and reduce morning home systolic blood pressure of hypertensive patients.
